# Lysine fatty acylation promotes lysosomal targeting of TNF-α

**DOI:** 10.1038/srep24371

**Published:** 2016-04-15

**Authors:** Hong Jiang, Xiaoyu Zhang, Hening Lin

**Affiliations:** 1 Department of Chemistry and Chemical Biology, Cornell University, Ithaca, NY 14853, USA; 2 Howard Hughes Medical Institute, Cornell University, Ithaca, NY 14853, USA

## Abstract

Tumor necrosis factor-α (TNF-α) is a proinflammation cytokine secreted by various cells. Understanding its secretive pathway is important to understand the biological functions of TNF-α and diseases associated with TNF-α. TNF-α is one of the first proteins known be modified by lysine fatty acylation (e.g. myristoylation). We previously demonstrated that SIRT6, a member of the mammalian sirtuin family of enzymes, can remove the fatty acyl modification on TNF-α and promote its secretion. However, the mechanistic details about how lysine fatty acylation regulates TNF-α secretion have been unknown. Here we present experimental data supporting that lysine fatty acylation promotes lysosomal targeting of TNF-α. The result is an important first step toward understanding the biological functions of lysine fatty acylation.

TNF-α is a proinflammation cytokine produced by various types of cells, including macrophages, lymphocytes, dendritic cells, and neurons^1^,^2^,^3^. It binds to TNF-α receptors and triggers signaling pathways that affect apoptosis and cell survival^4^,^5^. Dysregulation of TNF-α is associated with many inflammatory diseases, including rheumatoid arthritis, psoriatic arthritis, and inflammatory bowel disease^2^,^6^,^7^.

There are two protein forms of TNF-α, membrane TNF-α (mTNF-α, 26 kD) and secreted TNF-α (sTNF-α, 17 kD)^8^. mTNF-α is a type II transmembrane protein, with a single transmembrane domain linking the intracellular N-terminal domain and extracellular C-terminal domain. It is synthesized at the endoplasmic reticulum (ER) and transported to plasma membrane through Golgi and recycling endosomes^9^,^10^,^11^,^12^. At the plasma membrane, it is quickly digested by the metalloprotease TNF-α converting enzyme (TACE) to release the C-terminal sTNF-α^13^,^14^. The native mTNF-α and sTNF-α are trimers^15^,^16^,^17^,^18^,^19^ and both of them have been demonstrated to have biological activities and bind to TNF-α receptors^4^,^8^.

Because TNF-α has important immune functions, there has been significant research interest in the regulation of TNF-α. TNF-α is known to be regulated by many protein posttranslational modifications. In addition to proteolytic cleavage by TACE, proteases SPPL2a and SPPL2b were found to cleave the intramembrane part of mTNF-α and promote the release of the intracellular domain to induce the expression of pro-inflammatory cytokine interleukin-12^20^,^21^. TNF-α is also modified by O- and N-glycosylation, cysteine fatty acylation, and lysine fatty acylation. It was reported that human TNF-α has O-glycosylation at Ser80^22^ and mouse TNF-α has N-glycosylation at Asn86^23^. Recently, glycosylation at Ser80 of rat TNF-α was shown to regulate the sorting of TNF-α into mast cell granules^24^. Cysteine fatty acylation was found on human TNF-α at Cys30^25^. Fatty acylation of Cys30 is thought to facilitate the association of TNF-α with plasma membrane and its integration into lipid raft^26^. Although lysine fatty acylation on mTNF-α at Lys19 and Lys20 was discovered in early 1990s^27^, the effect of this modification on TNF-α was only recently elucidated. Lysine fatty acylation on TNF-α decreases its secretion and this modification is regulated by SIRT6, a nicotinamide adenine dinucleotide (NAD^+^)-dependent deacylase^28^. This was the first demonstration that lysine fatty acylation can regulate protein secretion. However, how lysine fatty acylation affects TNF-α secretion has been unknown. Here we demonstrate that lysine fatty acylation helps to target TNF-α to the lysosomes.

## Results

### Lysine fatty-acylated TNF-α localizes to large membrane vesicles

Previously we demonstrated that lysine fatty acylation downregulated TNF-α secretion (the production of sTNF-α)^28^. The secretion of sTNF-α in Sirt6 knockout (KO) or knockdown (KD) cells was lower than that in control cells. When Sirt6 KD cells were treated with palmitic acid to increase the level of lysine fatty acylation on TNF-α, the secretion efficiency of TNF-α in Sirt6 KD cells decreased even more dramatically^28^.

**Figure 1:**
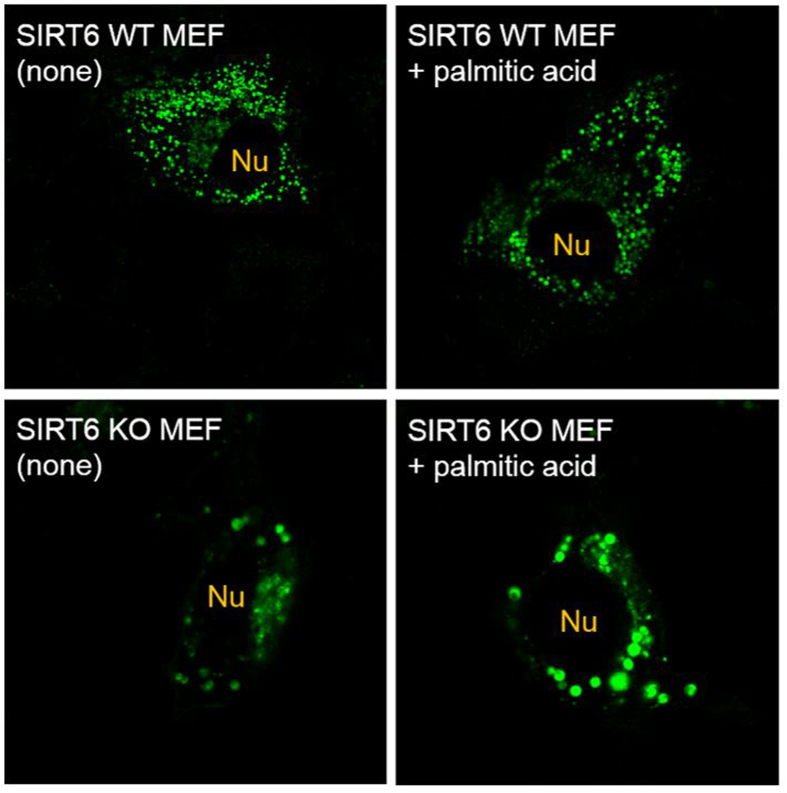
Confocal images of TNF-α_WT_GFP in WT and Sirt6 KO MEF cells treated with or without palmitic acid. “Nu” indicates the position of the nucleus.

In order to find out how lysine fatty acylation affects TNF-α secretion, we overexpressed wildtype (WT) TNF-α with a C-terminal green fluorescent protein (GFP), termed TNF-α_WT_GFP, in WT and Sirt6 KO MEF cells and examined the cellular localization of TNF-α. Confocal imaging results (Fig. 1) showed that TNF-α localized to distinct vesicles near the nucleus and throughout the cytosol in WT MEF cells. The size of TNF-α containing vesicles remained small when cells were treated with palmitic acid. While in Sirt6 KO MEF cells, TNF-α existed in large vesicles, which became even larger when the cells were treated with palmitic acid.

### Lysine fatty acylation retains TNF-α in lysosomes

**Figure 2:**
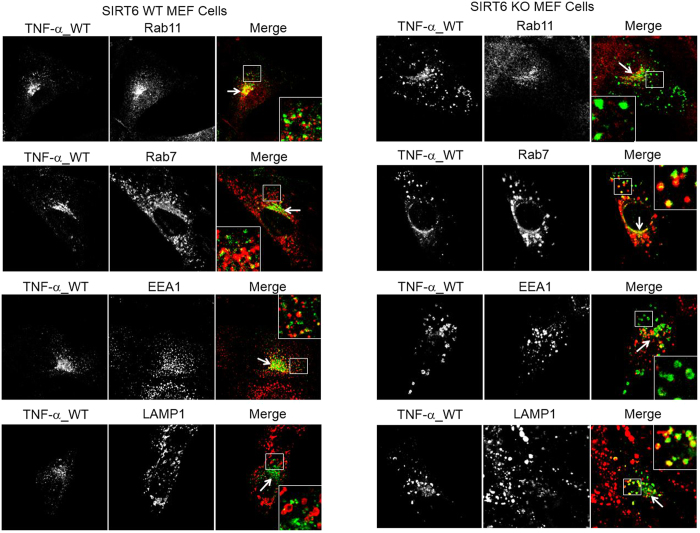
Co-localization of TNF-α_WT_GFP with different intracellular organelle markers in WT and Sirt6 KO MEF cells treated with 50 μM palmitic acid. Rab11, Rab7, EEA1 and LAMP1 are the protein markers of recycling endosomes, late endosomes, early endosomes and lysosomes, respectively. Arrows point to perinuclear regions while squares highlight nucleus-distal regions containing TNF-α_WT_GFP.

**Figure 3:**
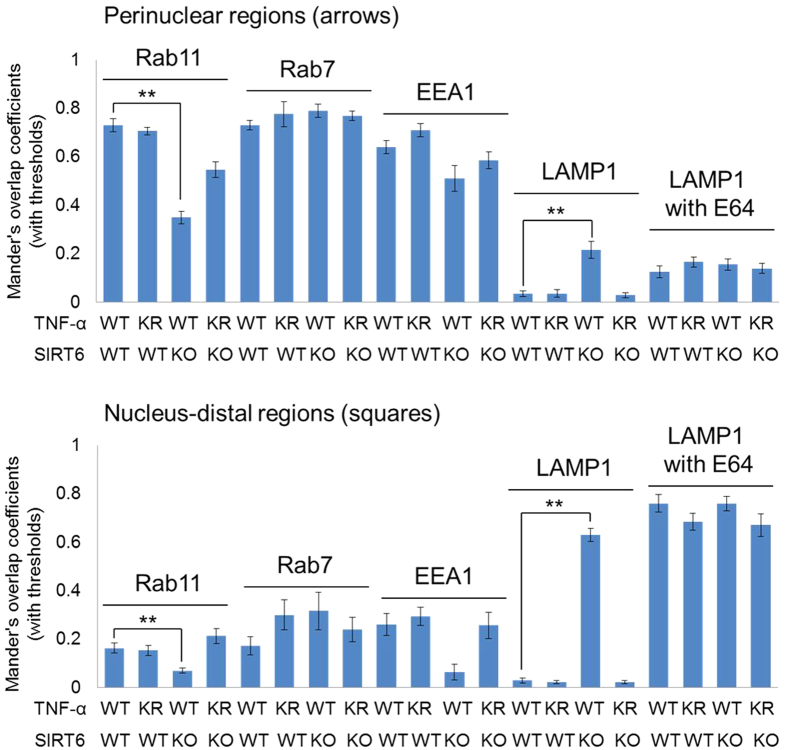
Overlap coefficients of TNF-α_WT_GFP and TNF-α_KR_GFP with Rab11 (recycling endosomes), Rab7 (late endosomes), EEA1 (early endosomes), and LAMP1 (lysosome). Co-localization was quantified using ImageJ. Each bar is the mean ± SD (error bars) for three images. Two asterisks indicate significance with *p* < 0.01.

We next set out to find out the identities of the TNF-α containing vesicles in both WT and Sirt6 KO MEF cells. We examined the co-localization of TNF-α with protein markers of various organelles involved in protein secretion^29^, including Rab11 (recycling endosome marker), EEA1 (early endosome marker), Rab7 (late endosome marker), and LAMP1 (lysosome marker) (Fig. 2). In Sirt6 WT cells, TNF-α co-localized with Rab11, EEA1 and Rab7 but not LAMP1 (Fig. 2, quantification of co-localization is shown in Fig. 3), in both perinuclear regions (arrows) and nucleus-distal regions (squares). These results indicated that TNF-α resided in recycling endosomes, early endosomes, and late endosomes but not in lysosomes in WT MEF cells. This is consistent with early report that TNF-α is secreted via the recycling endosomes^9^. In contrast, in Sirt6 KO cells, TNF-α co-localized with the lysosomal marker LAMP1 very well, suggesting that TNF-α localized more in lysosomes in Sirt6 KO cells (Fig. 2, right, and Fig. 3). In the Sirt6 KO cells, we still found co-localization of TNF-α with Rab11, EEA1, and Rab7. However, co-localization with the recycling endosome marker, Rab11, was significantly decreased in Sirt6 KO cells compared to that in Sirt6 WT cells (Figs 2 and 3). This suggested that the TNF-α localization in the recycling endosomes was decreased by Sirt6 KO, most likely due to the increased localization of TNF-α in the lysosomes.

**Figure 4:**
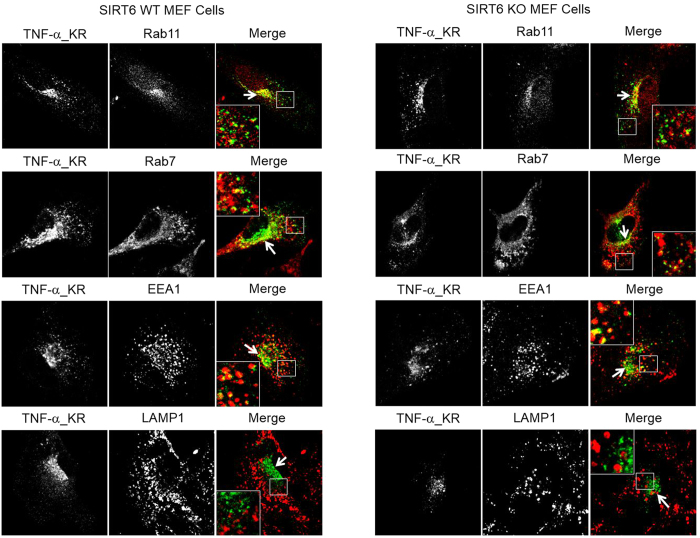
Co-localization of mutant TNF-α_KR_GFP with different protein markers in WT and Sirt6 KO MEF cells cultured in the presence of 50 μM palmitic acid. Rab11, Rab7, EEA1 and LAMP1 are the protein markers of recycling endosomes, late endosomes, early endosomes, and lysosomes, respectively.

The different localizations of TNF-α in WT and Sirt6 KO cells could be caused by the different levels of lysine fatty acylation on TNF-α or by differences in the secretory pathway. In order to find out whether the different localizations of TNF-α in WT and Sirt6 KO MEF cells were caused by differences in TNF-α lysine fatty acylation, we further examined the localization of the TNF-α mutant, TNF-α_KR, in which both Lys19 and Lys20 were mutated to Arg (Fig. 4). TNF-α_KR is defective in lysine fatty acylation and thus should not be affected by SIRT6-catalyzed defatty-acylation. In both WT and Sirt6 KO cells, the localization of TNF-α_KR was similar to that of TNF-α_WT (Fig. 4, quantification of co-localization is shown in Fig. 3). In particular, TNF-α_KR did not co-localize with LAMP1 in either WT or Sirt6 KO cells (Figs 3 and 4). This confirmed that localization of TNF-α_WT in the lysosomes of Sirt6 KO MEF cells was directly caused by lysine fatty acylation on TNF-α_WT.

**Figure 5:**
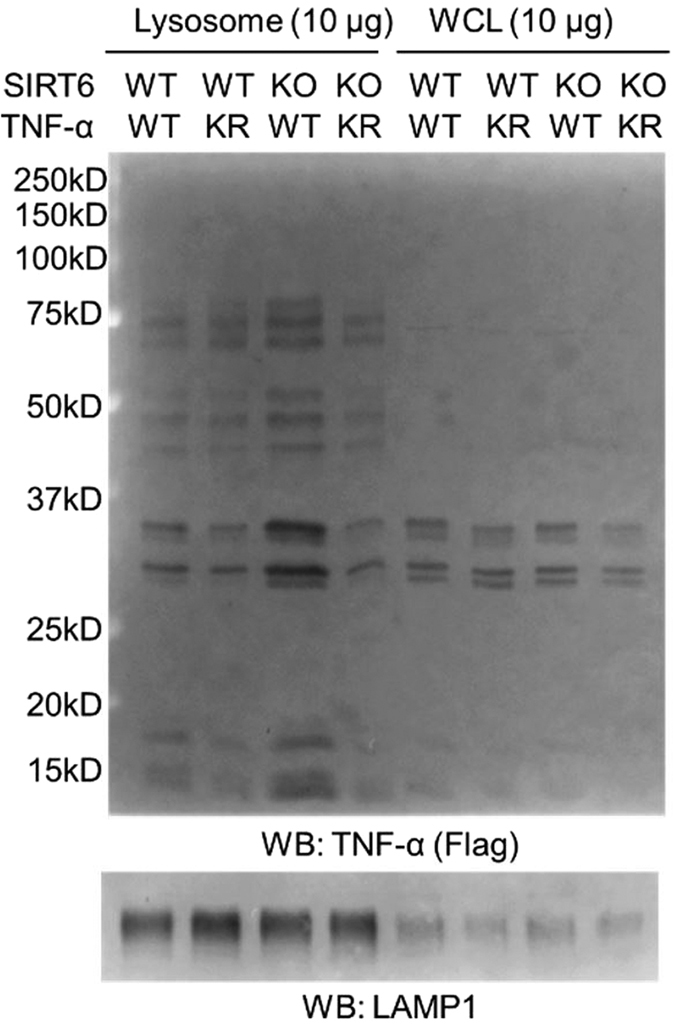
Lysosome fractionation of WT and Sirt6 KO MEF cells to determine the localization of TNF-α WT and the KR mutant. LAMP1 was used as the lysosome marker. WCL: whole cell lysates. For each fraction, equal amount of protein (10 μg) was loaded.

To further confirm the results obtained with confocal imaging, we also employed lysosome fractionation to determine the lysosomal localization of TNF-α WT and KR in WT and Sirt6 KO MEF cells. Consistent with the imaging data, more TNF-α WT was found in the lysosomal fraction of Sirt6 KO cells than WT cells (Fig. 5). In contrast, the levels of TNF-α KR mutant were similar in the lysosomal fractions of WT and Sirt6 KO MEF cells (Fig. 5).

### A portion of unacylated TNF-α is also targeted to lysosome for degradation

**Figure 6:**
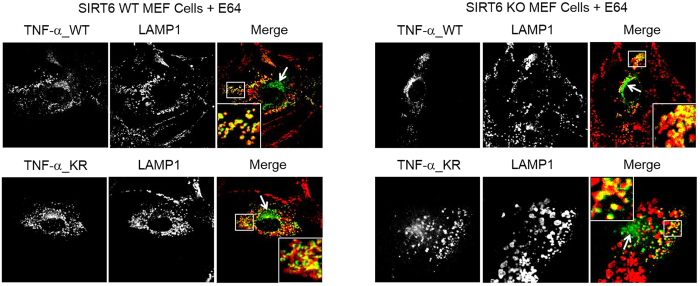
Co-localization of TNF-α_WT_GFP and TNF-α_KR_GFP with the lysosomal marker, LAMP1, in WT and Sirt6 KO MEF cells after treatment with E64.

Although no TNF-α was observed in the lysosomes in Sirt6 WT cells (Fig. 2), we wondered whether unacylated TNF-α molecules in Sirt6 WT cells were also targeted to lysosomes but they were either degraded quickly or exit the lysosome quickly. To investigate this, we treated cells with E64^30^, a lysosomal protease inhibitor, and then examined the localization of TNF-α_WT_GFP and TNF-α_KR_GFP via confocal imaging (Fig. 6). Interestingly, when treated with E64, we observed significant amount of co-localization of TNF-α_WT and TNF-α_KR with LAMP1 (Figs 3 and 6) in both WT and Sirt6 KO MEF cells.

**Figure 7:**
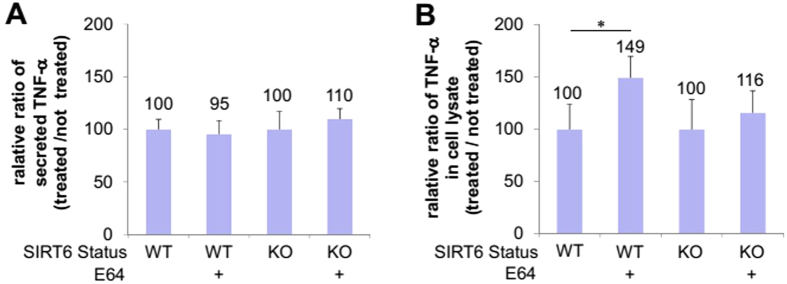
The effect of E64 on TNF-α secretion. (**A**) Relative (with E64 treatment versus without E64 treatment) levels of sTNF-α from WT and Sirt6 KO MEF cells. (**B**) Relative (with E64 treatment versus without E64 treatment) levels of intracellular mTNF-α in WT and Sirt6 KO MEF cells. Each bar is the mean ± SD (error bars) for three independent experiments. One asterisk indicates significance with *p* < 0.05.

These results suggest that unacylated TNF-α can also be targeted to the lysosome, but they are degraded very quickly. If TNF-α molecules are targeted to the lysosome and degraded, we would predict that E64 would not affect the amount of TNF-α secreted. However, it is also possible that the TNF-α molecules are targeted to lysosome as part of the normal secretory pathway (for example, cleavage of the transmembrane domain by lysosome proteases can actually create the sTNF-α). In that case, we would predict that E64 would decrease the amount of sTNF-α secreted. To differentiate the two possibilities, we measured the sTNF-α secretion in WT and Sirt6 KO MEF cells with or without E64 treatment (Fig. 7). The secretion of sTNF-α was not significantly affected by E64 treatment in either WT or Sirt6 KO cells (Fig. 7A). The only consequence of E64 treatment was that there was significantly increased TNF-α_WT inside of WT MEF cells (49% increase, Fig. 7B), which was consistent with the increased localization of TNF-α_WT_GFP in the lysosomes of WT MEF cells under E64 treatment (Fig. 6). Thus, the secretion results suggest that in WT cells, a certain percentages of TNF-α molecules are delivered to lysosomes, but they are quickly degraded and cannot be seen in the lysosomes without E64 treatment. In the Sirt6 KO cells, more TNF-α molecules are targeted to the lysosomes, which allows the detection of TNF-α in the lysosomes even without E64 treatment.

### Endogenous TNF-α in THP-1 cells is also targeted to lysosome by lysine fatty acylation

**Figure 8:**
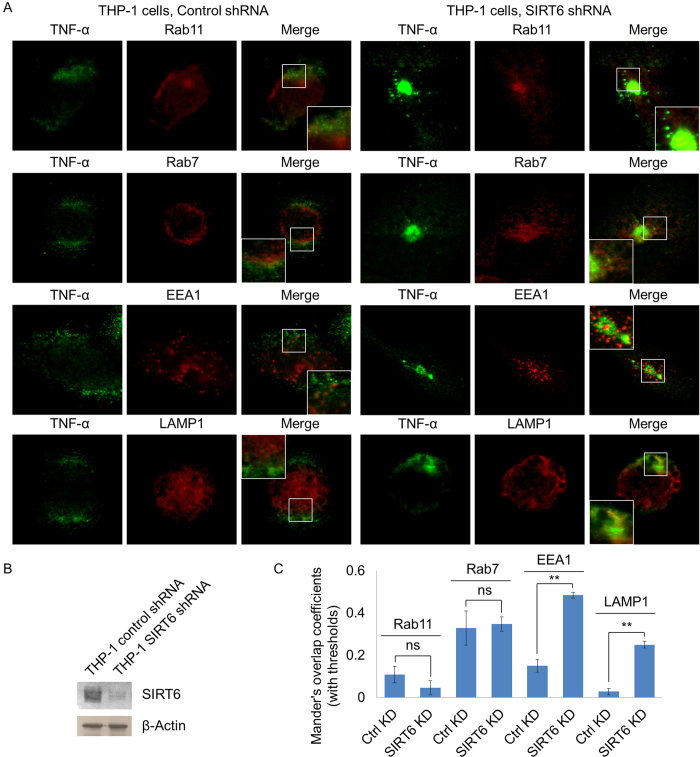
Co-localization of endogenous TNF-α with different intracellular organelle markers in THP-1 cells with control shRNA or Sirt6 shRNA. (**A**) Confocal imaging of endogenous TNF-α, Rab11 (recycling endosome marker), Rab7 (late endosome marker), EEA1 (early endosome marker), and LAMP1 (lysosome marker) in THP-1 cells. (**B**) Western blot of endogenous SIRT6 in THP-1 cells with control shRNA or Sirt6 shRNA. (**C**) Overlap coefficients of TNF-α with Rab11, Rab7, EEA1, and LAMP1. Co-localization was quantified using coloc 2 plugin in ImageJ. Each bar is the mean ± SD (error bars) for three images. Two asterisks indicate significance with *p* < 0.01.

All the above experiments were carried out with over-expressed TNF-α. To make sure endogenous TNF-α are also regulated similarly by SIRT6 and lysine fatty acylation, we examined the localization of endogenous TNF-α in THP-1 cells with control or Sirt6 knockdown (Fig. 8B). Similar to the localization in MEF cells, endogenous TNF-α existed in large vesicles in THP-1 cells with Sirt6 knockdown (Fig. 8A). In THP-1 cells with control shRNA, endogenous TNF-α co-localized with EEA1, Rab11, Rab7, but very little co-localized with LAMP1 (Fig. 8A,C). When SIRT6 was knocked down, TNF-α showed significantly increased co-localization with lysosome marker LAMP1 (Fig. 8A,C). Different from the results obtained in MEF cells, TNF-α also showed increased EEA1 co-localization in SIRT6 knockdown THP-1 cells than that in control THP-1 cells (Fig. 8A,C). Overall, these results demonstrate that lysine fatty acylation also helps to target endogenous TNF-α to lysosomes in THP-1 cells.

## Discussion

Understanding the mechanism that regulates cytokine secretion is essential to understand and treat cytokine-associated diseases. There are canonical and non-canonical secretion pathways for various cytokines^10^,^11^,^12^. TNF-α is generally believed to be secreted via the canonical pathway, from the ER/Golgi to plasma membrane through recycling endosomes^10^,^11^,^12^. We previously reported that secretion of sTNF-α is regulated by reversible lysine fatty acylation^28^. Lysine fatty acylation was discovered over 20 years ago, and there are only a few mammalian proteins known to have this modification, including interleukin 1α and TNF-α^27^,^31^. Regulating TNF-α secretion is the only known physiological function of lysine fatty acylation. However, how lysine fatty acylation regulates the secretion of TNF-α has been unknown. Our current study now provides more in-depth insights into how lysine fatty acylation may affect protein secretion.

**Figure 9:**
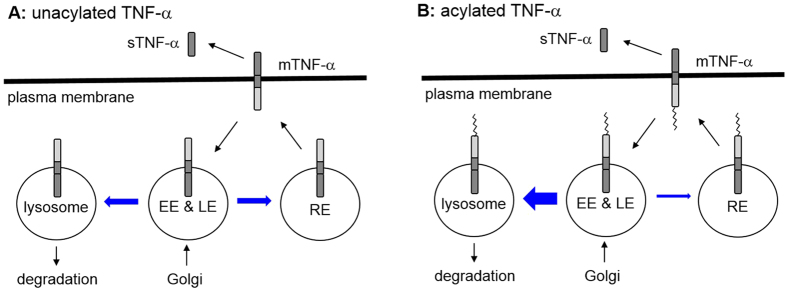
The model of TNF-α secretion and the effects of lysine fatty acylation. The thickness of the blue arrow indicates the relative amount of TNF-α that goes through that path. Lysine fatty acylation promotes the lysosomal targeting of TNF-α and the degradation of TNF-α.

The data presented here demonstrate that lysine fatty acylation helps to target TNF-α to the lysosomes for degradation and at the same time decrease its localization in the recycling endosome (Fig. 9), which explains why Sirt6 KO cells have decreased TNF-α secretion. The lysosomal localization of TNF-α in Sirt6 KO cells is directly determined by TNF-α lysine fatty acylation, as the TNF-α_KR mutant that cannot be lysine fatty-acylated is not localized in the lysosomes in Sirt6 KO cells. Some unfatty-acylated TNF-α molecules in Sirt6 WT cells are also targeted to the lysosomes, but they are quickly degraded and cannot be seen in the lysosomes unless a lysosomal protease inhibitor E64 is used.

The observation that TNF-α can only be observed in the lysosomes of Sirt6 KO cells may be caused by a combination of increased lysosomal targeting and resistance to lysosomal degradation. Given that lysine fatty acylation decreases TNF-α secretion, we are certain that lysosome targeting is increased by lysine fatty acylation (if lysine fatty acylation only increases resistance to lysosomal protease cleavage, the overall secretion would not be affected by lysine fatty acylation). However, we cannot rule out that lysine fatty acylation also increases resistance to lysosomal degradation. Whether TNF-α degradation by lysosomal proteases is affected by lysine fatty acylation awaits further studies.

Interestingly, it was previously reported that hypoxia reduced TNF-α secretion and enhanced co-localization of TNF-α with LAMP1^32^. Our results here may explain how TNF-α lysosomal localization increased under hypoxia condition^32^. It is possible that the level of lysine fatty acylation on TNF-α increases in hypoxia because cellular NAD^+^ level decreases in hypoxia^33^, which reduces SIRT6 enzymatic activity and increases lysine fatty acylation and thus lysosomal localization of TNF-α^28^.

It is not clear at this point whether lysine fatty acylation is a general mechanism for lysosomal targeting or not as only one defatty-acylation substrate for SIRT6 has been reported thus far. This question will be addressed when more defatty-acylation substrates of SIRT6 or other sirtuins are discovered in the future.

## Methods

### Reagents

Palmitic acid, E64, phorbol 12-myristate 13-acetate (PMA) and lipopolysaccharides from *Escherichia coli* 0111:B4 (LPS) were purchased from Sigma-Aldrich (St. Louis, MO). Anti-Flag antibody conjugated with horseradish peroxidase (A8592) was purchased from Sigma-Aldrich. Rat monoclonal anti-mouse LAMP1 antibody (1D4B) was purchased from BD Biosciences (San Jose, CA). Goat anti-rat IgG conjugated with Alexa Fluor 647 was purchased from Life Technologies (Grand Island, NY). Rabbit monoclonal anti-Rab11 antibody (D4F5), rabbit monoclonal anti-Rab7 antibody (D95F2), rabbit monoclonal anti-EEA1 antibody (C45B10), rabbit monoclonal anti-SIRT6 antibody (D8D12), goat anti-rabbit IgG conjugated with Alexa Fluor 647, and ProLong Gold antifade reagent with DAPI were purchased from Cell Signaling Technology (Danvers, MA). Anti-human TNF-α antibody (MAB610) was purchased from R&D systems (Minneapolis, MN). Human TNF-α ELISA kit was purchased from eBioscience. ECL plus western blotting detection reagent and Lysosome enrichment kit were purchased from Thermo Fisher Scientific Inc. SIRT6 shRNA lentiviral plasmid (pLKO.1-puro vector) was purchased from Sigma, the sequence of shRNA was: TRCN0000378253 (ccggcagtacgtccgagacacagtcctcgaggactgtgctcggacgtactgtttttg). Plasmid of pEGFP-N1 vector was a kind gift from Professor Barbara A. Baird.

### Cloning of TNF-α_WT and TNF-α_KR into pCMV4A and pEGFP-N1 vector

For construction of the expression vector for wild-type human TNF-α protein (TNF-α_WT) or the double lysine mutant (TNF-α_KR, K19R, K20R) with N-terminal Flag or C-terminal EGFP tag, full-length TNF-α_WT or TNF-α_KR cDNA^28^ were generated by PCR and inserted into pCMV4A vector at EcoRV and XhoI sites or pEGFP-N1 vector at Xho I and BamH I sites.

### Cells and Cell Transfection

THP-1 cells were maintained in Roswell Park Memorial Institute Medium (RPMI) supplemented with 10% heat-inactivated fetal bovine serum (Life Technologies). WT and Sirt6 KO MEF cells^28^ were maintained in Dulbecco’s Modified Eagle Medium (DMEM) containing glucose and L-glutamine (Life Technologies, Grand Island, NY) supplemented with 10% heat-inactivated fetal bovine serum. Human Embryonic Kidney (HEK) 293T cells were maintained in DMEM supplemented with 10% heat-inactivated fetal bovine serum. The TNF-α_WT and TNF-α_KR in pCMV4A or pEGFP-N1 vector were transfected into WT and Sirt6 KO MEF cells (50% confluency) using FuGene 6 transfection reagent (Promega, Madison, WI) according to the manufacture’s protocol.

### Generation of stable SIRT6 knockdown THP-1 cells

SIRT6 shRNA plasmid, pCMV-dR8.2, and pMD2.G were co-transfected into HEK 293T cells. After 24 hr, the medium was collected to infect the THP-1 cells. After infection for 6 hr, the medium was changed to fresh medium and the cells were cultured for 72 hr. Then 1.5 μg/mL of puromycin was added into the cells for at least three generations to select stable SIRT6 knockdown cells.

### Confocal imaging of TNF-α_WT_GFP or TNF-α_KR_GFP in WT and Sirt6 KO MEF cells

After WT and Sirt6 KO MEF cells were transfected with TNF-α_WT_GFP or TNF-α_KR_GFP for 12 hr, cells were incubated with fresh medium containing 50 μM palmitic acid or 50 μM E64 or DMSO (as control) for 6 hr. Then cells were washed with 1 mL 1× PBS buffer and fixed with 1 mL 4% paraformaldehyde (PFA) for 15 min at room temperature. After washing cells 3 times with 1 mL 1× PBS buffer, cells were permeabilized with 1 mL Saponin-BSA solution (0.1% Saponin with 3% BSA in 1× PBS buffer with 0.02% NaN_3_) for 1 hr at room temperature and then incubated with primary antibody (against Rab7, Rab11, EEA1 or LAMP1) solution (100 μL in Saponin-BSA solution) at 4 °C for 12 hr. After washing 4 times with 1 mL Saponin-BSA solution, cells were incubated with the secondary antibody (goat anti-rabbit or anti-rat IgG conjugated with Alexa Fluor 647) solution (100 μL in Saponin-BSA solution) for 2 hr at room temperature. After washing 4 times with 1 mL Saponin-BSA solution, cells were incubated with 250 μL ProLong Gold antifade reagent with DAPI for confocal imaging experiments. For quantification of colocalization, Mander’s overlap coefficients in images were determined using ImageJ coloalization plugin Intensity Correlation Analysis^34^ with manual setting of thresholds.

### Confocal imaging of endogenous TNF-α_in THP-1 cells

THP-1 cells (with control shRNA or SIRT6 shRNA) were treated with PMA (200 ng/mL) for 24 hr. Then cells were cultured in fresh RMPI medium with palmitic acid (50 μM) and LPS (1 μg/mL) for 4 hr. After washing with 1 mL 1× PBS buffer, the cells were fixed with 1 mL 4% PFA for 15 min at room temperature. The cells were then washed twice with 1× PBS buffer and permeabilized with 1 mL Saponin-BSA solution (0.1% Saponin with 3% BSA in 1× PBS buffer with 0.02% NaN_3_) for 1 hr at room temperature. The cells were then incubated with the primary antibodies (against TNF-α, Rab7, Rab11, EEA1 or LAMP1) in Saponin-BSA solution at 4 °C for 12 hr. After washing 3 times with 1 mL Saponin-BSA solution, the cells were incubated with the secondary antibodies (goat anti-rabbit or anti-rat IgG conjugated with Alexa Fluor 647) solution in Saponin-BSA solution for 1 hr at room temperature. After washing 3 times with 1 mL Saponin-BSA solution, confocal imaging experiments were performed. For quantification of co-localization, Mander’s overlap coefficients in images were determined using ImageJ coloc 2 plugin with manual setting of thresholds.

### Lysosome fractionation of WT and Sirt6 KO MEF cells

After WT and Sirt6 KO MEF cells were transfected with TNF-α WT or TNF-α KR for 24 hr, the cells were collected. Lysosome enrichment kit was used to fractionate the lysosome fraction. Flag western blot was then performed to determine the TNF-α expression in lysosome fraction and total lysates. The chemiluminescence signal in membrane was recorded after developing in ECL plus western blotting detection reagents using Typhoon 9400 Variable Mode Imager (GE Healthcare Life Sciences).

### Secretion of TNF-α_WT and TNF-α_KR from WT and Sirt6 KO MEF Cells treated with or without E64

After transient transfection of TNF-α_WT or TNF-α_KR^28^ for 6 hr, WT and Sirt6 KO MEF cells were further incubated with fresh medium containing 10 μM E64 or DMSO (as control) for 12 hr. Then the culture medium and cells were collected separately for detection of TNF-α using human TNF-α ELISA kit. Three independent experiments were carried out.

### Statistical Analysis

Data were expressed as mean ± SD (standard deviation*, shown as error bars*). Differences were examined by Student’s *t test* between two groups. P value below 0.05 was attributed to significance and represented by one *asterisk*. P value below 0.01 was attributed to most significance and represented by double *asterisks*.

## Additional Information

**How to cite this article**: Jiang, H. *et al.* Lysine fatty acylation promotes lysosomal targeting of TNF-α. *Sci. Rep.* **6**, 24371; doi: 10.1038/srep24371 (2016).
